# Brazilin isolated from *Caesalpinia sappan* L. acts as a novel collagen receptor agonist in human platelets

**DOI:** 10.1186/1423-0127-20-4

**Published:** 2013-01-25

**Authors:** Yi Chang, Steven Kuan-Hua Huang, Wan-Jung Lu, Chi-Li Chung, Wei-Lin Chen, Shun-Hua Lu, Kuan-Hung Lin, Joen-Rong Sheu

**Affiliations:** 1 Department of Anesthesiology, Shin Kong Wu Ho-Su Memorial Hospital, 95 Wen-Chang Rd, Taipei, 11101, Taiwan; 2 School of Medicine, Fu-Jen Catholic University, 510 Zhong-Zheng Rd, Taipei, 24205, Taiwan; 3 Division of Urology, Department of Surgery, Chi-Mei Medical Center, 901 Zhong-Hua Rd, Tainan, 71004, Taiwan; 4 Department of Pharmacology, College of Medicine, Taipei Medical University, 250 Wu-Hsing St, Taipei, 11031, Taiwan; 5 Graduate Institute Medical Sciences, College of Medicine, Taipei Medical University, 250 Wu-Hsing St, Taipei, 11031, Taiwan; 6 Division of Pulmonary Medicine, Department of Internal Medicine, Taipei Medical University Hospital, 252 Wu-Hsing St, Taipei, 11031, Taiwan; 7 School of Respiratory Therapy, College of Medicine, Taipei Medical University, 250 Wu-Hsing St, Taipei, 11031, Taiwan; 8 Central Laboratory, Shin Kong Wu Ho-Su Memorial Hospital, 95 Wen-Chang Rd, Taipei, 11101, Taiwan

**Keywords:** Brazilin, Collagen receptors, Lyn phosphorylation, Occlusion time, Platelet activation

## Abstract

**Background:**

Brazilin, isolated from the heartwood of *Caesalpinia sappan* L., has been shown to possess multiple pharmacological properties.

**Methods:**

In this study, platelet aggregation, flow cytometry, immunoblotting analysis, and electron spin resonance (ESR) spectrometry were used to investigate the effects of brazilin on platelet activation *ex vivo*. Moreover, fluorescein sodium-induced platelet thrombi of mesenteric microvessels was also used in *in vivo* study.

**Results:**

We demonstrated that relatively low concentrations of brazilin (1 to 10 μM) potentiated platelet aggregation induced by collagen (0.1 μg/ml) in washed human platelets. Higher concentrations of brazilin (20 to 50 μM) directly triggered platelet aggregation. Brazilin-mediated platelet aggregation was slightly inhibited by ATP (an antagonist of ADP). It was not inhibited by yohimbine (an antagonist of epinephrine), by SCH79797 (an antagonist of thrombin protease-activated receptor [PAR] 1), or by tcY-NH2 (an antagonist of PAR 4). Brazilin did not significantly affect FITC-triflavin binding to the integrin α_IIb_β_3_ in platelet suspensions. Pretreatment of the platelets with caffeic acid phenethyl ester (an antagonist of collagen receptors) or JAQ1 and Sam.G4 monoclonal antibodies raised against collagen receptor glycoprotein VI and integrin α_2_β_1_, respectively, abolished platelet aggregation stimulated by collagen or brazilin. The immunoblotting analysis showed that brazilin stimulated the phosphorylation of phospholipase C (PLC)γ2 and Lyn, which were significantly attenuated in the presence of JAQ1 and Sam.G4. In addition, brazilin did not significantly trigger hydroxyl radical formation in ESR analysis. An in vivo mouse study showed that brazilin treatment (2 and 4 mg/kg) significantly shortened the occlusion time for platelet plug formation in mesenteric venules.

**Conclusion:**

To the best of our knowledge, this study provides the first evidence that brazilin acts a novel collagen receptor agonist. Brazilin is a plant-based natural product, may offer therapeutic potential as intended anti-thrombotic agents for targeting of collagen receptors or to be used a useful tool for the study of detailed mechanisms in collagen receptors-mediated platelet activation.

## Background

**Figure 1:**
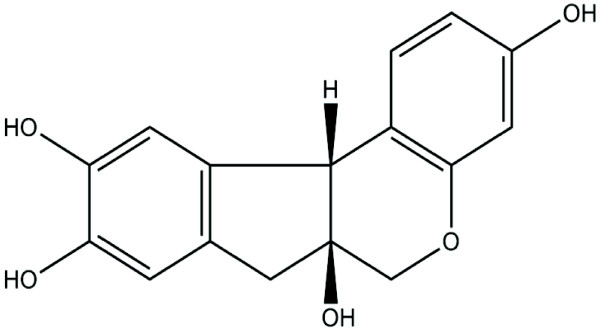
Chemical structure of brazilin.

Brazilin (7,11*b*-dihydrobenz*b*indeno[1,2-*d*pyran-3,6a,9,10(6H)-tetrol) is the major component isolated from the heartwood of *Caesalpinia sappan* L. (Leguminosae) (Figure 1). *C. sappan* has long been widely used as an oriental traditional or folk medicine. It is considered an analgesic and anti-inflammatory agent and has been used to treat emmeniopathy, sprains, and convulsions [1]; it has also been used to treat diabetic complications [2] and to improve blood circulation [3]. Extracts of *C. sappan* have been shown to exert various pharmacological effects, including anti-hypercholesterolemia, sedation, and depression of the central nervous system [4]. In addition, it is an anti-hepatitis B surface antigen (HBsAg) [5] and lowers the motility of human sperm [6]. Brazilin is also used as a natural red pigment for histological staining [7]. Several studies have shown that the anti-hyperglycemic [8], anti-hepatotoxic [9], and anti-inflammatory effects of brazilin are caused by the inhibition of inducible nitric oxide synthase (NOS) in macrophage cells [10], and vasorelaxation induced by the activation of NOS in endothelial cells [4].

Intravascular thrombosis is associated with several cardiovascular diseases. The initiation of an intraluminal thrombosis is thought to involve platelet adherence and aggregation. During normal circulation, platelets do not aggregate. However, when a blood vessel is damaged, platelets adhere to the disrupted surface and release biologically active constituents that induce aggregation [11]. Resting (circulating) platelets are anuclear cells, discoid in shape, which originate from megakaryocytes in the bone marrow. Platelets may be activated by various physiological or pharmacological agents. Physiological agents include thrombin, collagen, ADP, platelet-activating factor (PAF), and epinephrine, whereas pharmacological agents include calcium ionophores and cyclic endoperoxide analogues. Upon activation, the platelets lose their discoid shape and become more spherical, extending long, spiky pseudopods and bulky surface protrusions [12]. The various agonists are thought to exert their effects by interacting with specific receptors on platelet membranes. Platelet activation plays a crucial role in numerous cardiovascular and cerebrovascular disorders.

Until this study, no data had been published on the effect of brazilin in platelet activation. One study reported that brazilin significantly inhibited thrombin-, collagen-, and ADP-induced platelet aggregation in washed rat platelets [13]. By contrast, our preliminary study showed that brazilin potentiated or stimulated platelet aggregation in washed human platelets. This discrepancy might result from specie-specific characteristics of platelets. We thus systematically examined the influence of brazilin in human platelets ex vivo and in platelet plug formation in vivo. The findings were used to characterize the mechanisms of brazilin-mediated activation in human platelets.

## Methods

### Materials

ATP, caffeic acid phenethyl ester (CAPE), collagen (type I, bovine achilles tendon), heparin, fluorescein sodium, yohimbine, prostaglandin E_1_ (PGE_1_), arachidonic acid (AA), ADP, thrombin, U73122, 5,5-dimethyl-1 pyrroline N-oxide (DMPO), and bovine serum albumin (BSA) were purchased from Sigma Chem. (St Louis, MO). SCH79797 and trans-cinnamoyl-YPGKF-NH2 (tcY-NH_2_) were obtained from TOCRIS Bioscience (Ellisville, MIS). Anti-glycoprotein (GP) VI (JAQ1) and anti-integrin α_2_β_1_ (Sam.G4) monoclonal antibodies (mAbs) were obtained from Emfret Analytics (Würzburg, Germany); anti-phospholipase Cγ2 (PLCγ2) and anti-phospho (Tyr^759^) PLCγ2 polyclonal antibodies (pAbs) from Cell Signaling (Beverly, MA); anti-Lyn and anti-phospho Lyn pAbs from Santa Cruz (Santa Cruz, CA). The Hybond-P polyvinylidene difluoride (PVDF) membrane, an enhanced chemiluminescence (ECL) Western blotting detection reagent and analysis system, horseradish peroxidase (HRP)-conjugated donkey anti-rabbit IgG, and sheep anti-mouse IgG were from Amersham (Buckinghamshire, UK). The brazilin was purchased from MP Biomedical (Solon, OH). We dissolved the brazilin in 0.5% dimethyl sulfoxide (DMSO) and stored it at 4°C until use.

### Preparation of human platelet suspensions

Human platelet suspensions were prepared as previously described [14]. This study conformed to the principles outlined in the *Helsinki Declaration*, and human volunteers gave informed consent. In brief, blood was collected from healthy human volunteers who had taken no medicine during the preceding 2 wk, and was mixed with acid-citrate-dextrose (ACD) (9:1, v/v). After centrifugation, the supernatant (platelet-rich plasma; PRP) was supplemented with PGE_1_ (0.5 μM) and heparin (6.4 IU/ml) and incubated for 10 min. The mixture was then centrifuged at 500 *g*; thereafter, the platelets were washed and suspended in a Tyrode’s solution containing BSA (3.5 mg/ml). The final concentration of Ca^+2^ in the Tyrode’s solution was 1 mM.

### Platelet aggregation

A turbidimetric method was applied to measure platelet aggregation [14], using a Lumi-Aggregometer (Payton, Canada). Platelet suspensions (3.6 × 10^8^ cells/ml) were pretreated with or without reagents for 3 min, followed by the addition of brazilin or various agonists to trigger platelet activation. The reaction was allowed to proceed for at least 6 min, and the extent of aggregation was expressed in light-transmission units.

### Flow cytometric analysis

Fluorescence-conjugated triflavin, an α_IIb_β_3_ disintegrin, was prepared as previously described [11]. Platelet suspensions (3.6 × 10^8^ cells/ml) were preincubated with brazilin (25 and 50 μM) or a solvent control (0.5% DMSO) for 3 min, followed by the addition of 2 μl of a solution of FITC-triflavin (2 μg/ml). The suspensions were then assayed for fluorescein-labeled platelets, using a flow cytometer (Beckman Coulter, Miami, FL). Data were collected from 50,000 platelets per experimental group, and the platelets were identified by their characteristic forward and orthogonal light-scattering profiles. All experiments were repeated at least 4 times to ensure reliability.

### Immunoblotting

Washed platelets (1 × 10^9^ cells/ml) were preincubated with reagents for 3 min, followed by the addition of agonists to trigger platelet activation. The reaction was stopped, and platelets were immediately re-suspended in 200 μl of lysis buffer. Samples containing 80 μg of protein were separated using a 12% sodium dodecylsulfate polyacrylamide gel electrophoresis (SDS-PAGE); proteins were electrotransferred by semidry transfer (Bio-Rad, Hercules, CA). Blots were blocked with TBST (10 mM Tris-base, 100 mM NaCl, and 0.01% Tween 20) containing 5% BSA for 1 h and then probed with various primary antibodies. Membranes were incubated with HRP-linked anti-mouse IgG or anti-rabbit IgG (diluted 1:3000 in TBST) for 1 h. Immunoreactive bands were detected using an ECL system. Bar graphs depicting quantitative ratios were produced by scanning the reactive bands and quantifying their optical density using videodensitometry (Bio-profil; Biolight Windows Application V2000.01; Vilber Lourmat, France).

### Measurement of hydroxyl radicals by electron spin resonance (ESR) spectrometry

The ESR method used a Bruker EMX ESR spectrometer as described previously [15]. In brief, platelet suspensions (3.6 × 10^8^ cells/ml) were incubated with brazilin (25 and 50 μM), collagen (1 μg/ml) or a solvent control (0.5% DMSO) for 3 min. The reaction was allowed to proceed for 5 min, followed by the addition of DMPO (100 μM) for the ESR study.

### Fluorescein sodium-induced platelet thrombi in mesenteric microvessels of mice

As described previously [14], mice were anesthetized, and an external jugular vein was cannulated with PE-10 so that dye and medication could be administered by an intravenous (i.v.) bolus. A segment of the small intestine was placed onto a transparent culture dish for microscopic observation. Venules (30 to 40 μm) were selected for irradiation to produce a microthrombus. Using the epi-illumination system, light from a 100-W mercury lamp was passed through a B-2A filter (Nikon, Tokyo, Japan) with a DM 510 dichromic mirror (Nikon). Wavelengths below 520 nm had been eliminated from the filtered light, which was used to irradiate a microvessel; the area of irradiation was approximately 100 μm in diameter on the focal plane. Various dosages of brazilin (2 and 4 mg/kg) or an isovolumetric solvent control (0.5% DMSO) was administered 1 min after fluorescein sodium (15 μg/kg) administration. Five minutes after administration of the fluorescein sodium, irradiation by filtered light and the video timer were simultaneously begun, and platelet aggregation was observed on a television monitor. The time lapse for inducing thrombus formation leading to the cessation of blood flow was measured.

### Statistical analysis

The experimental results are expressed as the means **±** S.E.M. and are accompanied by the number of observations. Paired Student’s *t*-test was used to determine significant differences in the in vivo studies of platelet plug formation. The other experiments were assessed by the method of analysis of variance (ANOVA). If this analysis indicated significant differences among the group means, then each group was compared using the Newman-Keuls method. A *p* value of less than 0.05 was considered statistically significant.

## Results

### The influence of brazilin on platelet aggregation in washed human platelets

**Figure 2:**
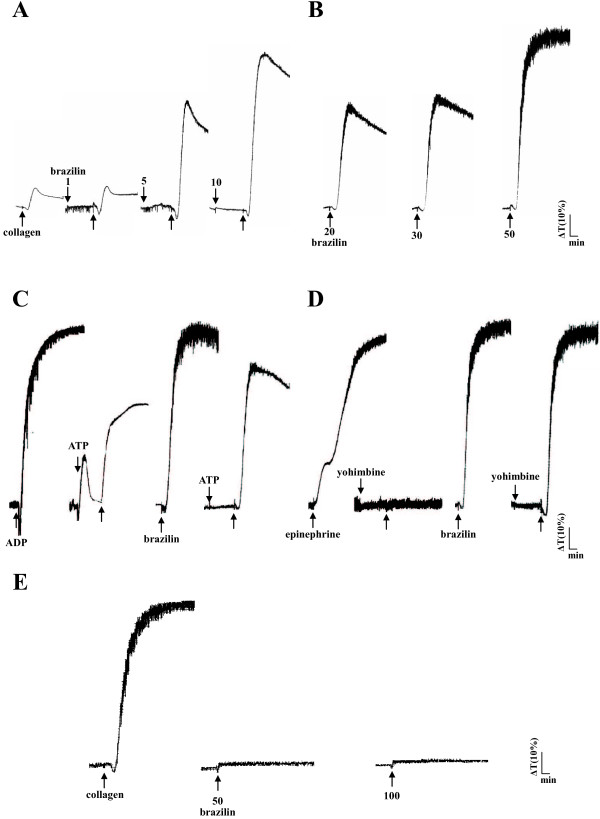
**Effect of brazilin in potentiating or stimulating human platelet aggregation in washed platelets or platelet-rich plasma.** Washed platelets (3.6 × 10^8^/ml) were incubated with brazilin (1 to 50 μM), either with (**A**) or without (**B**) the addition of collagen (0.1 μg/ml) to trigger platelet aggregation. In additional experiments, washed platelets (3.6 × 10^8^/ml) were preincubated with (**C**) ATP (50 μM) or (**D**) yohimbine (5 μM); this was followed by the addition of ADP (20 μM), brazilin (50 μM), or epinephrine (10 μM) to trigger platelet aggregation. (**E**) Furthermore, platelet-rich plasma was incubated with collagen (1 μg/ml) or brazilin (50 to 10 μM) to trigger platelet aggregation. Aggregation profiles (**A** to **E**) are representative examples of 4 similar experiments.

**Figure 3:**
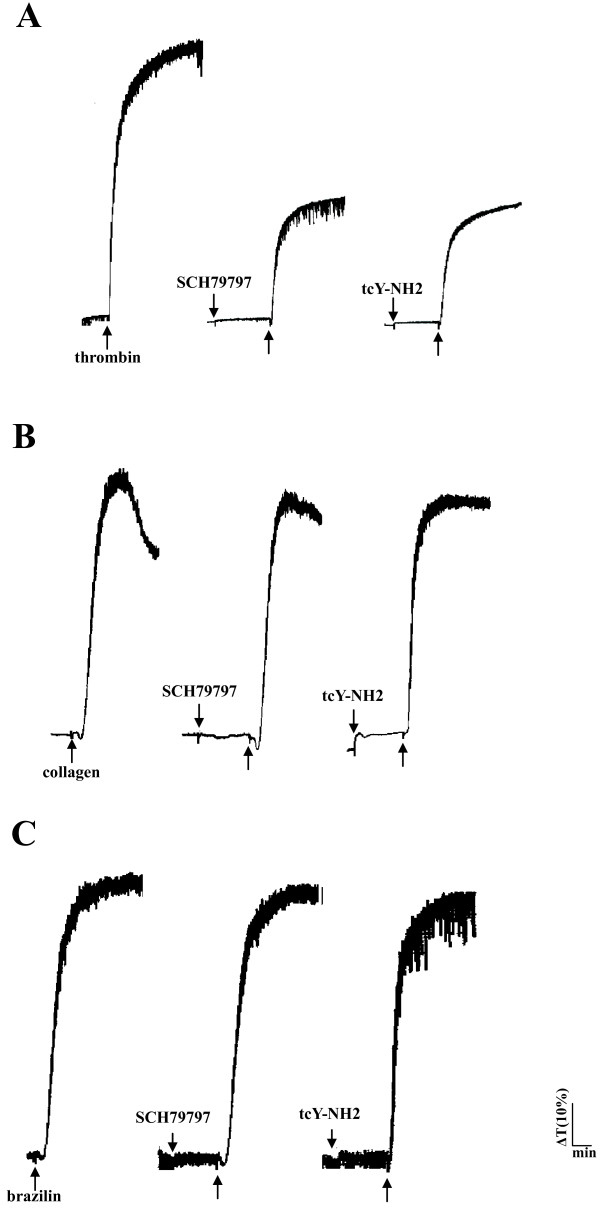
**The influence of antagonists of thrombin receptors in brazilin-mediated platelet aggregation.** Washed platelets (3.6 × 10^8^/ml) were preincubated with SCH79797 (1 μM) or tcY-NH2 (100 μM); this was followed by the addition of (**A**) thrombin (0.5 U/ml), (**B**) collagen (1 μg/ml), or (**C**) brazilin (50 μM) to trigger platelet aggregation. Aggregation profiles (**A** to **C**) are representative examples of 4 similar experiments.

Low concentrations of brazilin (1, 5, and 10 μM) potentiated platelet aggregation in a concentration-dependent manner induced by a sub-threshold concentration of collagen (0.1 μg/ml) in washed human platelets (Figure 2A). At higher concentrations (20, 30, and 50 μM), brazilin directly triggered platelet aggregation in a concentration-dependent manner (Figure 2B). A high dose of brazilin (50 μM) was equally potent in inducing platelet aggregation compared with collagen (1 μg/ml) (data not shown). As shown in Figure 2C, ATP (50 μM), an antagonist to ADP on platelets, inhibited platelet aggregation stimulated by ADP (20 μM) more effectively than that stimulated by brazilin (50 μM). Yohimbine (5 μM), an α_2_-adrenoceptor antagonist, inhibited platelet aggregation stimulated by epinephrine (10 μM), but not by brazilin (50 μM) (Figure 2D). However, brazilin (50 μM) did not significantly induce platelet aggregation in PRP even at a higher concentration of 100 μM (Figure 2E), indicating that brazilin might have high binding ability with protein in plasma. Furthermore, we also found that there are no significant differences in the platelet number between the control (0.5% DMSO) and brazilin-treated values (control, 1376.8 ± 284.1 × 10^6^/ml vs. 2 mg/kg brazilin, 1159.2 ± 383.4 × 10^6^/ml, *n*=5, *p* > 0.05; control, 1252.0 ± 310.6 × 10^6^/ml vs. 4 mg/kg brazilin, 1020.4 ± 328.7 × 10^6^/ml, *n*=5, *p* > 0.05) during the administration of various doses of brazilin (2 and 4 mg/kg) into the tail vein of mice (ICR strain) for 10 min. In addition, SCH79797 (1 μM), a thrombin protease-activated receptor (PAR) 1 antagonist, and tcY-NH2 (100 μM), a PAR 4 antagonist, both markedly diminished platelet aggregation stimulated by thrombin (0.5 U/ml) (Figure 3A), but not by collagen (1 μg/ml) (Figure 3B). Neither SCH79797 (1 μM) nor tcY-NH2 (100 μM) significantly affected platelet aggregation stimulated by brazilin (50 μM) (Figure 3C). These results indicated that brazilin-induced platelet aggregation was not mediated even partially by ADP receptors, α_2_-adrenoceptors, or thrombin PAR receptors on the platelet membranes.

### Collagen receptors involved in brazilin-induced platelet aggregation

**Figure 4:**
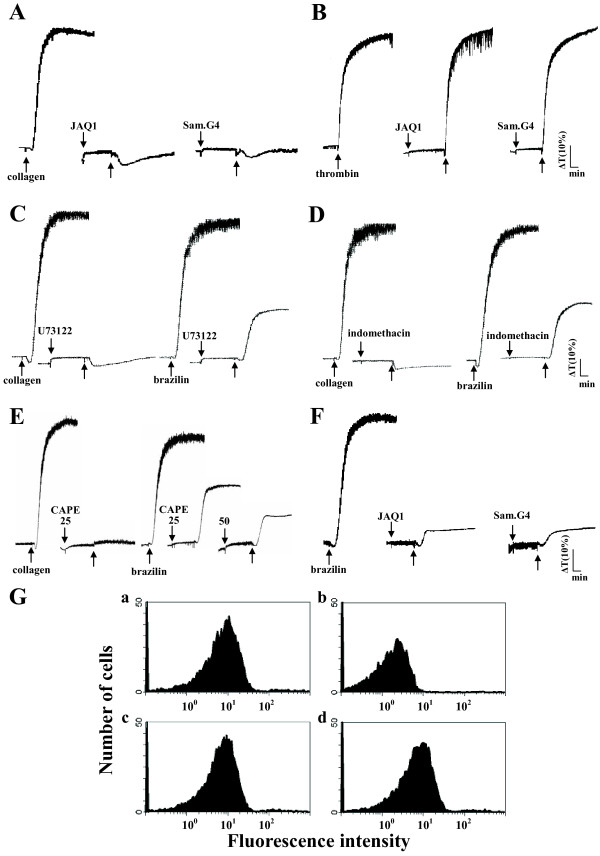
**The influence of antagonists of collagen receptors and integrin α_IIb_β_3_ in brazilin-mediated platelet aggregation.** Washed platelets (3.6 × 10^8^/ml) were preincubated with JAQ1 (4 μg/ml), Sam.G4 (4 μg/ml), U73122 (10 μM), indomethacin (25 μM), or caffeic acid phenethyl ester (CAPE; 25 and 50 μM). This was followed by the addition of (**A** and **C-E**) collagen (1 μg/ml), (**B**) thrombin (0.5 U/ml), or (**C**-**F**) brazilin (50 μM) to trigger platelet aggregation. (**G**) The solid line represents the fluorescence profiles of FITC-triflavin (a) in the absence of brazilin as a positive control; (b) in the presence of 5 mM EDTA as a negative control; (c) in the presence of 25 μM brazilin; or (d) in the presence of 50 μM brazilin. Thereafter, 2 μg/ml FITC-triflavin was added in each case. Profiles (**A** to **G**) are representative examples of 4 similar experiments.

GP VI and integrin α_2_β_1_ are the main collagen receptors involved in platelet adhesion and aggregation [16]. Pretreatment with JAQ1 (4 μg/ml) and Sam.G4 (4 μg/ml), which are mAbs against GP VI and integrin α_2_β_1_ respectively, abolished platelet aggregation stimulated by collagen (1 μg/ml) (Figure 4A) but not by thrombin (0.5 U/ml) (Figure 4B). U73122 (10 μM), a PLC inhibitor, and indomethacin (25 μM), a cyclooxygenase inhibitor, both markedly diminished platelet aggregation stimulated by collagen (1 μg/ml) and brazilin (50 μM) (Figures 4C and 4D), indicating that brazilin triggers platelet aggregation perhaps mediate by thromboxane A_2_-dependent mechanisms as collagen did. Furthermore, caffeic acid phenethyl ester (CAPE; 25 and 50 μM) [17], an active component of propolis obtained from honeybee hives, acts as a collagen receptor antagonist; this compound abolished platelet aggregation stimulated by either collagen (1 μg/ml) or brazilin (50 μM) (Figure 4E). Pretreatment with either JAQ1 (4 μg/ml) or Sam.G4 (4 μg/ml) abolished platelet aggregation stimulated by brazilin (50 μM) (Figure 4F). These results showed that brazilin might activate platelet aggregation through collagen receptors on the platelet membranes.

Triflavin is an α_IIb_β_3_ disintegrin, which inhibits platelet aggregation by directly interfering with fibrinogen binding to the integrin α_IIb_β_3_[11]. We evaluated whether brazilin would bind directly to the platelet integrin α_IIb_β_3_, leading to interruption of platelet aggregation. Our results showed that the relative intensity of the fluorescence of 2 μg/ml FITC-triflavin bound directly to platelets was 55.2 ± 4.5 (Figure 4G, a). The fluorescent intensity was markedly reduced in the presence of 5 mM EDTA (negative control, 5.2 ± 0.6) (Figure 4G, b). Brazilin (25 and 50 μM) did not significantly affect FITC-triflavin binding to the integrin α_IIb_β_3_ in platelet suspensions (25 μM, 55.1 ± 5.2; 50 μM, 54.3 ± 4.5) (Figure 4G, c and d). These results showed that the stimulatory effect of brazilin on platelet aggregation did not affect integrin α_IIb_β_3_. Overall, our findings provide evidences that brazilin acts as a collagen receptor agonist.

### Brazilin stimulated PLCγ2 and Lyn activation through collagen receptors

**Figure 5:**
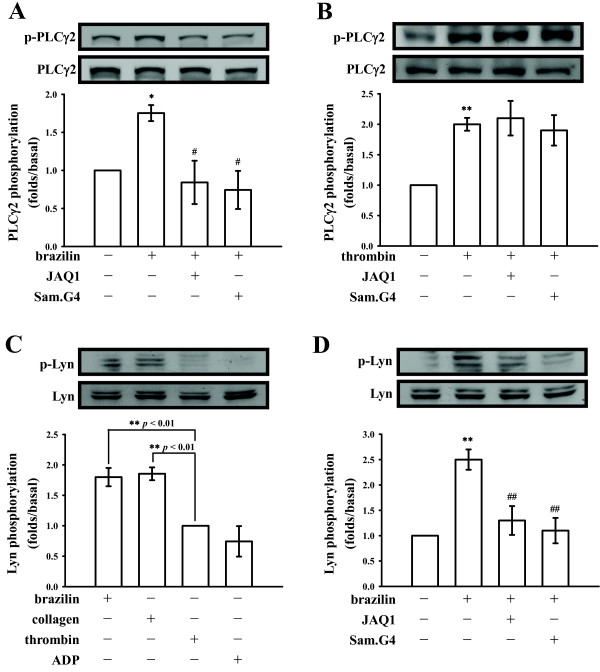
**Regulatory effects of antagonists of collagen receptors in brazilin-mediated phospholipase C (PLC)γ2 and Lyn phosphorylation of washed platelets.** Washed platelets (1.2 × 10^9^/ml) were preincubated with JAQ1 (4 μg/ml) or Sam.G4 (4 μg/ml), followed by the addition of (**A, C-D**) brazilin (50 μΜ) or (**B-C**) thrombin (0.5 U/ml), collagen (1 μg/ml), ADP (20 μΜ to trigger platelet activation. The reactions were stopped by the addition of EDTA (10 mM). Cells were then collected and subcellular extracts were analyzed for (**A-B**) PLCγ2 and (**C-D**) Lyn phosphorylation by immunoblotting, as described in the “Materials and methods” section. Profiles (**A** to **D**) are representative examples of 4 similar experiments. Data are presented as the means ± S.E.M. (*n*=4); ^*^p<0.05 and ^**^p<0.01, compared to the solvent (0.5% DMSO)- or thrombin-treated group; ^#^*p<*0.05 and ^##^*p<*0.01, compared to the brazilin-treated group.

PLC hydrolyzes phosphatidylinositol 4,5-bisphosphate (PIP_2_) to generate 2 secondary messengers: inositol 1,4,5-trisphosphate (IP_3_) and diacylglycerol (DAG). These messengers trigger platelet activation [18]. The immunoblotting analysis showed that brazilin (50 μM) treatment resulted in marked phosphorylation of PLCγ2 compared with resting platelets (Figure 5A). Treatment with JAQ1 (4 μg/ml) or Sam.G4 (4 μg/ml) significantly attenuated this phosphorylation stimulated by brazilin (Figure 5A), but not by thrombin (0.5 U/ml) (Figure 5B). In addition, Lyn was specifically phosphorylated by brazilin (50 μM) and collagen (1 μg/ml), but not by thrombin (0.5 U/ml) or ADP (20 μM) (Figure 5C). Brazilin (50 μM) stimulated Lyn phosphorylation was diminished in the presence of JAQ1 (4 μg/ml) or Sam.G4 (4 μg/ml) (Figure 5D).

### Influence of brazilin in hydroxyl radical formation in vitro and platelet plug formation in microvessels of mice

**Figure 6:**
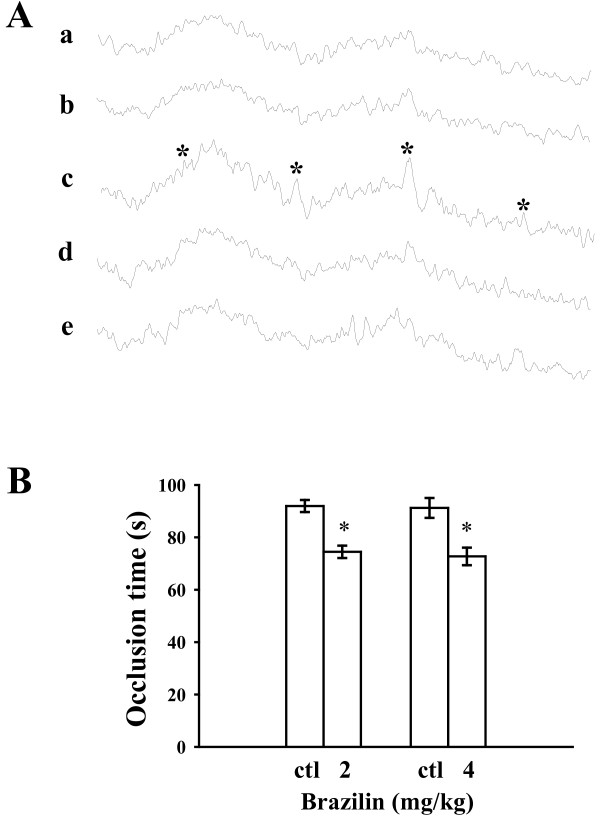
**Regulatory effects of brazilin in hydroxyl radical formation and the occlusion time required for thrombus formation in the mesenteric venules of mice. **(**A**) For the electron spin resonance (ESR) study, washed platelets were incubated with (a) Tyrode’s solution only (resting group), or platelets were incubated with (b) the solvent control (0.5% DMSO), (c) collagen (1 μg/ml), (d) brazilin (25 μM), or (e) brazilin (50 μM) to trigger hydroxyl radical formation. Asterisk (*) indicates the formation of hydroxyl radical. (**B**) Mice were administered either brazilin (2 and 4 mg/kg) or isovolumetric 0.5% DMSO (ctl; solvent control). Thereafter, mesenteric venules were selected for irradiation to induce microthrombus formation. The bar graphs (**A**) present the means ± S.E.M. of the occlusion time (s) for inducing platelet plug formation (n=5). ^*^p < 0.05 compared with the individual solvent control.

A typical ESR signal of hydroxyl radical (OH^●^) formation was triggered in collagen-activated platelets compared to resting platelets or 0.5% DMSO-treated platelets (Figures 6A, a, b, and c). However, treatment with brazilin (25 and 50 μM) did not significantly trigger hydroxyl radical formation (Figures 6A, d and e).

Our observation of thrombus formation in the microvessels of mice pretreated with fluorescein sodium (15 μg/kg) showed that the required occlusion time was approximately 90 s. When brazilin (2 and 4 mg/kg) was administered after pretreatment with fluorescein sodium, the occlusion time was significantly shorter compared with the solvent controls (occlusion time for 2 mg/kg brazilin was 74.4 ± 2.4 s compared with 91.9 ± 2.3 s for 0.5% DMSO; *n*=5, *p <* 0.05; for 4 mg/kg brazilin, occlusion time was 72.7 ± 3.4 s compared with 91.2 ± 3.8 s for 0.5% DMSO; *n*=5, *p <* 0.05) (Figure 6B). These results indicated that brazilin stimulated platelet plug formation in vivo.

## Discussion

In this study, up to our knowledge, this is a novel finding that brazilin, a plant-based natural product acts as a collagen receptor agonist induce platelet activation, other than that some collagen receptor agonists purified from the snake venoms [19,20].

Platelets are activated by a variety of physiological stimuli (e.g., thrombin, collagen, ADP, epinephrine, and PAF). These agonists are thought to exert their effects by interacting with specific receptors on the platelet membranes. The primary effects of agonists may be enhanced by secondary effects caused by the synthesis of thromboxane A_2_ (TxA_2_) from the arachidonic acid (AA) or by the secretion of ADP from the dense granules in platelets. ADP binds to 2 major purinergic receptors (P2Y_1_ and P2Y_12_), which play an important role in potentiating platelet activation induced by other aggregating agonists [21]. Therefore, ATP, an antagonist to ADP, might affect platelet aggregation stimulated by other agonists, including brazilin (Figure 2C).

Thrombin is one of the most potent activators of platelets and its role in promoting thrombus formation has been clearly established. Thrombin activates platelets through multiple cell-surface receptors, including the GP Ib/V/IX complex and the PARs [12]. Of the 4 known PAR isoforms, PAR1, PAR3, and PAR4 constitute the active thrombin receptors on human platelets [22]. PAR1 and PAR4 are essential for thrombin-induced human platelet activation [23]. Furthermore, epinephrine could induce platelet aggregation in the presence of sub-physiological calcium concentrations, as occurs in citrated plasma [24]. Aggregation as monitored in the light transmission aggregometer occurs without preceding shape change (disc to sphere transformation) (Figure 2D). Platelets possess stimulatory α_2_-adrenoceptors and inhibitory β-adrenoceptors; in most individuals the α_2_-adrenoceptors predominate.

Platelets adhere to the connective tissue protein collagen, with a resulting change in shape and the release of granules. Adhesion is partly dependent on the release of ADP and TxA_2_, whereas aggregation is entirely dependent on the release thereof [21]. The matrix protein collagen is present in the vascular subendothelium and vessel wall, and acts as a substrate for platelet adhesion; it is also an endogenous platelet activator. Among the platelet receptors known to interact directly with collagen, integrin α_2_β_1_ (GP Ia/IIa) and GP VI [19] appear to play a key role and have recently gained the attention of researchers. GP VI is widely recognized as a requisite factor for the formation of platelet aggregates on a collagen surface under blood flow [25]. Integrin α_2_β_1_ is another major collagen receptor on endothelial cells and platelets. In cells expressing integrin α_2_β_1_, many signals (including tyrosine phosphorylation and matrix remodeling) are activated after cell adhesion to collagen [26]. Recent findings suggest that integrin α_2_β_1_ and GP VI might contribute to the overall processes of platelet adhesion and activation [19,27,28].

GP VI is a platelet membrane protein with a molecular weight of 62 kDa. It has been identified as a physiological collagen receptor and belongs to a membrane of the immunoglobulin superfamily, which forms a complex with the Fc receptor γ-chain (FcRγ) containing immunoreceptor tyrosine-based activation motifs (ITAM) and is phosphorylated by Src-family kinases such as Fyn and Lyn [16,25]. Tyrosine kinases (Fyn and Lyn) are involved in GP VI-dependent activation and might phosphorylate the FcRγ [29]. Fyn and Lyn were shown to bind to the Pro-rich domain of the GP VI cytoplasmic tail in platelets [30], suggesting that the GP VI-dependent activation mechanism might be similar to that of the cytokine receptors. In this process, receptor-bound tyrosine kinases (such as Src) phosphorylate the cytoplasmic tails of receptors when the receptors become associated with each other through ligand binding. This phosphorylation will initiate the signal transduction pathway. In platelets, cross-linking of the GP VI/FcRγ complex would enable the GP VI-bound Fyn or Lyn to move to a position close enough to FcRγ that it would catalyze the phosphorylation of FcRγ ITAM. In turn, this triggers the phosphorylation of downstream signals, including the linker for activation of T-cells (LAT), leading to the activation of a kinase cascade (i.e., PLCγ_2_).

Our previous study [17] showed that the antiplatelet activity of CAPE might involve direct interference with the binding of collagen to its specific receptors on the platelet membrane. The current study showed that CAPE markedly inhibited brazilin-induced platelet aggregation. Furthermore, brazilin markedly stimulated platelet aggregation and PLCγ_2_ and Lyn phosphorylation. All these reactions were significantly diminished by JAQ1 (anti-GP VI mAb) and Sam.G4 (anti-integrin α_2_β_1_ mAb). Interestingly, we also found that the relative fluorescence intensity of the FITC-collagen (1 μg/ml) bound directly to platelets was 11.7 ± 1.9 (*n*=4) and hence the fluorescent intensity was markedly reduced in the presence of 1 μg/ml collagen (1.6 ± 1.4, *n*=4); however pretreatment with brazilin (25, 50, and 100 μM) showed a significant increase in the relative fluorescence intensity of FITC-collagen (25 μM, 33.8 ± 13.9; 50 μM, 38.4 ± 10.6; 100 μM, 61.8 ± 9.8; *n*=4) (data not shown). These results suggest that brazilin may act at the allosteric site to display allosteric agonism on collagen receptors, and subsequently enhances both the affinity and efficacy of collagen towards its binding sites. A similar model has been proposed in G-protein-coupled receptors and predicts that allosteric ligands bind to a topographically distinct site on a receptor to modulate orthosteric ligand affinity and/or efficacy [31]. A study also reported that some allosteric ligands can enhances both affinity and efficacy, and it displays allosteric agonism [31]. Therefore, we speculate that brazilin may serve as an allosteric ligand for collagen receptors in platelets. Overall, these results provided evidence that the stimulation of platelet activation by brazilin might be the result of direct stimulation of collagen receptors on the platelet membrane. However, our experiments did not rule out the possibility that other as-yet-unidentified mechanisms might be involved in brazilin-mediated platelet activation.

Reactive oxygen species (i.e., hydrogen peroxide and hydroxyl radicals) derived from platelet activation might amplify platelet reactivity during *in vivo* thrombus formation. Free radical species act as secondary messengers that increase cytosolic Ca^2+^ during the initial phase of platelet activation processes [15]. It is also evident that some of the hydrogen peroxide produced by platelets is converted into hydroxyl radicals, as platelet aggregation can be inhibited by hydroxyl radical scavengers [15]. In the present study, we found that brazilin did not significantly induce hydroxyl radical formation as compared with the collagen-stimulated platelets, indicating that brazilin may have a differential characterization on free radical formation apart from acting as the collagen receptor agonist in platelets. Following an injury to the endothelial cells, exposure of sub-endothelial collagen provides the major trigger to initiate platelet adhesion and aggregation at the site of injury. This is followed by arterial thrombus formation [11]. When platelets aggregate, they release a number of substances including TxA_2_ and ADP, both of which strengthen the platelet activation processes. He *et al*. [27] showed that integrin α_2_β_1_-deficient mice exhibited delayed thrombus formation following carotid artery injury. This result was consistent with the previously reported correlation between high levels of integrin α_2_β_1_ expression and increased risk for thrombosis involving the coronary and cerebral vessels [32,33]. Nieswandt *et al*. [34] reported that mice depleted of GP VI were completely protected from lethal collagen-induced pulmonary thromboemboli. Similarly, our study showed that brazilin potentiated platelet plug formation in the mesenteric venules of rats. Activated platelets also contribute to enhance the assembly and activity of two major coagulation factor complexes which facilitates coagulation and thrombus stabilization. Therefore, the coagulation factors may be involved in brazilin shortened the occlusion time in vivo.

## Conclusions

In conclusion, the key finding of this study was that brazilin acts as a collagen receptor agonist. However, the detailed mechanisms of brazilin-mediated signaling events in platelet activation require further investigation. Brazilin is a novel plant-based natural product, may offer therapeutic potential as intended anti-thrombotic agents for targeting of collagen receptors or to be used a useful tool for the study of detailed mechanisms in collagen receptors-mediated platelet activation.

## Abbreviations

AA: Arachidonic acid; ATP: Adenosine triphosphate; BSA: Bovine serum albumin; CAPE: Caffeic acid phenethyl ester; DAG: Diacylglycerol; GP: Glycoprotein; iNOS: Inducible nitric oxide synthase; PAF: Platelet-activating factor; PAR: Proteinase-activated receptor; PLC: Phospholipase C; PRP: Platelet-rich plasma; PGE_1_: Prostaglandin E_1_; TxA_2_: Thromboxane A_2_; TNF: Tumor necrosis factor.

## Competing interests

The authors declare that they have no competing interests.

## Authors’ contributions

YC and SKHH performed research and wrote the manuscript; WJL, CLC, WLC, and SHL performed the partial experiments and analyzed data; KHL and JRS conceived of the study and designed research. All authors read and approved the final manuscript.
